# Supplementary figures and images for: Estimation of neuron parameters from imperfect observations
Source: PLoS Comput Biol. 2020 Jul 16;16(7):e1008053. doi: 10.1371/journal.pcbi.1008053 (PMC7386621; doi:10.1371/journal.pcbi.1008053)

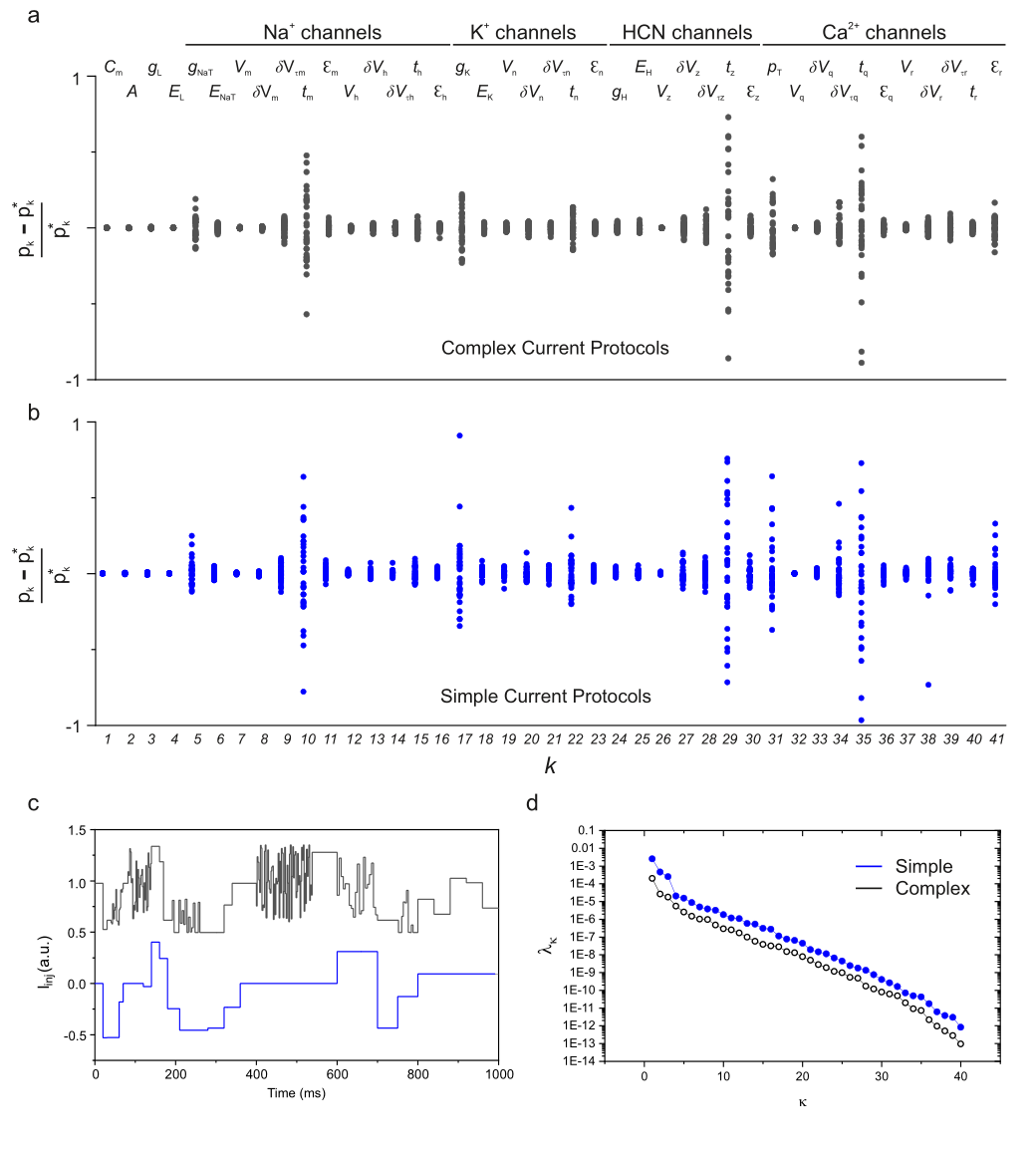

Supplement: S1 Fig — (a) Dispersion of extracted parameters of the RVLM neuron model in response to a complex current stimulation protocol (grey line). (b) Same as (a) for a simpler current protocol (blue line). (c) Complex (grey) and simple (blue) current protocols used to stimulate the neuron and to constraint the parameters obtained in (a) and (b). (d) Size-ranked eigenvalue spectra of the covariance matrices Σ^ of parameters estimated using the two current protocols in (c). (TIF) [file pcbi.1008053.s001.tif]
