# Supplementary material for: Estimation of neuron parameters from imperfect observations
Source: PLoS Comput Biol. 2020 Jul 16;16(7):e1008053. doi: 10.1371/journal.pcbi.1008053 (PMC7386621; doi:10.1371/journal.pcbi.1008053)
Supplement: Attachment — Submitted filename: paper-review-1.pdf [file pcbi.1008053.s002.pdf]

Reviewer comments:

Overall this article would be a worthwhile addition to the field of neuroscience and touches upon the common problem of parameter estimation for nonlinear models from noisy data. The paper is split into clear distinct sections and the figures are quite useful to gain an intuitive feel for the methods used. However, the authors should make their work slightly more accessible to a general audience and include more references to existing, different approaches to parameter estimation in the literature. Furthermore, the scope and applicability of the methods should be more clearly outlined, such that other readers in neuroscience can readily see in what circumstances this research is relevant. Sometimes the notation and the steps performed for each section are unclear. Nevertheless, these shortcomings should be relatively straightforward to address and does not require a radical overhaul of the paper.

In more detail:

Equation (1) – Are there any restrictions on the equations or the state variables for your approach? For example, can each state variable depend on all the other state variables, or are there restrictions on this?

Line 49 – Presumably "pairs of parameter sets" is meant here rather than two parameters from a given set.

Line 103 – Make it clear that x(0) refers to an initial condition of the state variables for the model.

Lines 119-120 – The description of the experimental error should be more closely related to the biophysics of the problem. For neurons there is channel noise, thermal fluctuations, flucutations in synaptic drive (if *in vivo*) along with measurement error coming from the equipment used to measure the neurons. Add some references to the different types of noise which are present in neurons and for electrophysiological measurement (e.g. Faisal, Selen, Wolpert, 2008: *Noise in the Nervous System*).

Line 121 – Presumably the additive noise at one time is independent from the noise at a different time. Briefly justify why this is a good assumption, as not all additive noise will necessarily have a white spectrum. Could your approach, in principle, accommodate temporally correlated noise?

Line 135 – Bold required for p_0^*.

Lines 147-148 – Add a further sentence for the general reader to intuitively explain the technical meaning of "sloppiness", as I would not assume that the majority readers would recognise this.

Lines 154-158, equation (6) – The noise entropy and this equation are not referred to afterwards in the paper, only two paragraphs before. Move this further up perhaps.

Line 175 – Why have you chosen a RVLM neuron model and not a different neuron model? As this is a test case for the methods you outline, explain what makes this model a good choice for this. This is touched upon in vague terms in lines 178-179 and later in the methods section, but you need to be more specific. The question that this explanation should answer to the reader is: what does your choice of model suggest about the applicability of your work to other neuronal models?

Lines 184, 194 – The useful membrane voltage that you have generated is now referred to as V_mem rather than V_use. Make this consistent with previous notation or adequately explain why the notation has been changed.

Lines 188-190 – Will the code used to perform the parameter search algorithm for the RVLM model upon acceptance and publication of the paper? Also, it is worth outlining that your four examples seem broadly applicable to any parameter estimation method that uses the same cost function. If some aspects are specific to an interior-point solver approach, please briefly state that this is the case.

Equation 9 – It is not explained before or immediately after what R is here (the number of realisations).

Line 229 – Throughout this paper “magnitude”, “noise intensity”, “noise amplitude” and “noise level” are used seemingly interchangeably for sigma. Be consistent and choose one of these to refer to sigma throughout, if indeed sigma refers to the same quantity in each section.

Lines 234-235 – How can one know in general which local minimum is the closest to the global minimum when a multitude of local minima may exist? Is some sort of heuristic in the Euclidean parameter distance used for this?

Line 241 - What is the meaning of a negative noise amplitude? Surely this cannot refer to the standard deviation of Gaussian-distributed noise, so what does sigma refer to in this case? Is it a constant scalar factor for the whole noise realisation?

Lines 248-249 – Can one safely assume that p_\sigma\zeta^* always lies inside the basin of attraction of the noise-free optimal parameter set? Is this guaranteed by the jump in delta p and delta c?

Line 257 – epsilon_z has not been introduced yet.

Lines 278-279 – What does it mean for the method without regularization to converge 67% of the time? Are there different initial conditions x(0) or initial parameter guesses? How many trials were performed for each case? Furthermore, why does the regularized method fail 6% of the time ? Is there an obvious pattern to the failures?

Do you have a comparison of the average computation time for the method without regularization to the method with regularization? One may ask whether it is quicker just to run the simulation without additive noise multiple times in the hope that the optimal parameter set is reached at least once.

Lines 319-320 – Presumably this is p_0^v for one particular noise realisation? Furthermore, p_0^v seems to represent the same concept as p_\sigma\zeta^* in the regularization section. The notation should be made consistent if this is indeed the case and the connection between these two sections in general should be made stronger.

Line 339 – Minor point: Takens’

Lines 350-352 – This claim needs a reference.

Line 380 – Elsewhere in the paper 94% probability was given for convergence.

Lines 380-382 – Do you have an intuitive idea on how small the experimental error would have to be in comparison to the additive noise used for regularization? It would be nice if this additive noise regularization could be applied to already noisy data. Briefly state why the task is "hopeless" when the errors are sufficiently large.

Sequential Monte Carlo (or particle filter) approaches have been used for parameter inference for nonlinear models from noisy neuron data (e.g. Huys and Paninski, 2009: *Smoothing of, and Parameter Estimation from, Noisy Biophysical Recordings* and Vavoulis, Straub, Aston and Feng, 2012: *A Self-Organizing State-Space-Model Approach for Parameter Estimation in Hodgkin-Huxley-Type Models of Single Neurons)*. How does your research relate to this?

Line 381 – Typo: "their"

Line 435 – Per area membrane capacitance

Fig 3a - Is the panel drawn here merely illustrative of the method, or does the curve actual represent how the cost function varies according to a specific parameter from the set (if the latter, which parameter)?

Figure 3e – This panel is just two numbers, it seems a bit redundant.

Figure 5a – The blue dots are not explained or referenced in the text. Are they other noise realisations of p_0^v?
