# Supplementary material for: Estimation of neuron parameters from imperfect observations
Source: PLoS Comput Biol. 2020 Jul 16;16(7):e1008053. doi: 10.1371/journal.pcbi.1008053 (PMC7386621; doi:10.1371/journal.pcbi.1008053)
Supplement: Attachment — Submitted filename: main.pdf [file pcbi.1008053.s003.pdf]

Review Estimation of neuron parameters from imperfect observations

April 29, 2020

The paper *Review Estimation of neuron parameters from imperfect observations* discusses the model parameter estimation of single neurons based on observed membrane potential. The authors introduce key challenges, namely observability, identifiability, local minima and ill-definition. Further on, the authors discuss two sources of errors, experimental errors and model errors and how they influence the global and local minima of the cost function by second-order expansion. They discuss very detailed the effect of experimental error and model error on location and orientation of minima. As a solution, the authors suggest a new method by scaling the experimental noise, which moves and suppresses local minima, making it more likely to identify the optimal parameters of the model. They present numerical results based on RVLM neurons where they demonstrate the improvements in parameter estimation by their proposed new method.

As a second part, the authors discuss the requirement to drive the model by a various external current to cover the full range of dynamical behaviour of the model. The authors study the parameter estimation and their correlation by varying the number of data points. They suggest a method of non-uniform sampling the time series to optimize parameter estimation and de-correlation while not increasing the number of data points. Authors support this by numerical experiments.

In summary, the authors provide a very detailed and extensive discussion of the topic. The reach of the study is limited by the assumption that the model $\mathbf{F}$ is known and only the model parameters $\mathbf{p}$ need to be estimated. In an experimental setting this would not be the case. It would help the understanding of the paper if the authors could mention this limitation. It was not part of this study to optimise parameter estimation if the data generating model is not equal to the fitted model as it would be with experimental data. As far as I understand is the entire study based on the assumption that experimental noise is $\sim iid\mathcal{N}(0, \sigma)$, additive and uncorrelated. I believe the consequences of this assumption could be discussed in more detailed and maybe also the effect of different types of noise. To me, the section describing the noise regularization to improve convergence is a bit unclear: The text says a specific noise realization is selected and this this noise realization is scaled in amplitude. Does this

not imply that the noise is already known¿ This section could be a little more detailed.
The manuscript is very clear and consistent when it comes to mathematical formulations. I believe the manuscript could benefit from an iteration of the text, especially for readers which are non-experts.
Overall, I think the paper covers an interesting topic in a very strict mathematical form. The manuscript could be improved by discussing the implications of the made assumptions as well as by improving the writing to make the text easier to understand and read for non-experts. Therefore, I reccomend a minor revision.

I would like to mention some specific points which could be changed to improve the manuscript:

1 Abstract

- The sentence `This regularization method increases the probability of finding the true parameter configuration from 67% to 94.3%.` is unclear since it is not defined what the true parameters are. Later on it is mentioned that it is meant finding the global minimum, but this could be mentioned here as well to avoid confusion.

2 Introduction

- Line 35: Would make it easier to understand if the $L + K$ state would be explicitly defined as the parameter state
- 71: Since the PDF is non Gaussian, could be helpful to also use percentiles or something which could deal with heavy tailed or other distributions.
- Line 74: Define `surfaces of constant misfit`
- Line 81: Sentence hard to understand

3 Results

- Line 123: Should the model errors also depend on $I(t)$?
- Line 139: The model has not been introduced, so this sentence is hard to understand. Also, what is exactly the sensitivity of the membrane voltage?
- Line 159: What is principal analysis?
- Line 241: How is negative noise amplitude defined in context of normal distributions? Should σ not be larger than zero?
- Line 280: Why does a random input I not work?

- Line 250: The parameter search was initialized given the ground truth. What happened if it was randomly initialized?

4 Methods

- Line 511: Units not consistent, if u is in volts, why is derivative of u unitless?
