# Supplementary material for: Estimation of neuron parameters from imperfect observations
Source: PLoS Comput Biol. 2020 Jul 16;16(7):e1008053. doi: 10.1371/journal.pcbi.1008053 (PMC7386621; doi:10.1371/journal.pcbi.1008053)
Supplement: Attachment — Submitted filename: Reply_reviewers.pdf [file pcbi.1008053.s004.pdf]

We are very grateful to the reviewers for their constructive feedback and the improvements they suggested on the manuscript. We reply to individual comments below. The corresponding changes made to the manuscript are indicated in red in the text.

Reply to Reviewer #1:

This fairly theoretical paper describes a process to estimate parameters of neuron models. The method is applied using surrogate data obtained from running a model of a rostral ventrolateral medulla neuron. It contains several techniques to find back the global optimal estimate of the parameters of the model in question. I think the paper is fairly well written, and rigorously describes the math behind the method.

However, in general I have two problems with the paper in its current form:

Firstly, although the method is described very rigorously, I find the results obtained rather less convincing. The method is only applied on a single model. For the results of that single model not many statistics are used. Quite some results are based on a single observation (as an example I'd give Fig 5). It would be good to show that the results are more robust over more models and/or more trials.

Secondly, a big drawback of this paper is that it is based on surrogate data. Over the years many theoretical papers have been published with methods to optimize neuron parameters, but not many of these are actually used in real situations. The reason is that there is a world of difference between surrogate data and real experimental data. Just adding some noise to the surrogate data doesn't really represent the real world problems. Therefore I'd strongly suggest that the authors consider at least to add an example where they apply their method to real experimental data. I do understand that of course in that case some analysis can't be performed, because one doesn't know the original parameters, but it still allows for an analysis of number of solutions found, etc.

REPLY 1: If we may, we answer the two connected points above in the reverse order.

Point 2: we have already extensively demonstrated the success of the interior point assimilation method for building conductance models of many “real world” neurons including:

- *songbird neurons* (Meliza et al., *Biol. Cyb.* **108**, 495 (2014), Nogaret et al., *Sci. Rep.* **6**, 32749 (2016), Wang et al., *IEEE Trans. Biomed. Devices* **11**, 1258 (2017)),
- *rodent hippocampal neurons* (CA1, CA3) (Abu-Hassan et al, *Nature Communications*, **10**, 5309 (2019))
- *rodent respiratory neurons* (Abu-Hassan et al, *Nature Communications*, **10**, 5309 (2019)).

Completed models have made predictions that were virtually identical to the experimentally observed oscillations of biological neurons under any stimulation protocol. It is the dispersion of parameters observed by us and other authors when assimilating real neurons that prompted the present study. As the referee points out, understanding the causes of this dispersion requires going back to surrogate data because twin experiments are the only way to determine the global solution and validate convergence of data assimilation.

Point 1: Biological neuron data have also been assimilated using two qualitatively different models and their variations to account for a variable numbers of ion channels:

- conductance models derived from the Hodgkin-Huxley type equations (Meliza et al., 2014; Nogaret et al., 2016)
- conductance models derived from the equations of CMOS microcircuits (Abu-Hassan et al., 2019; Wang et al., 2017)

Within each of these models, the complement of ion channels has been varied. In addition, the statistical analysis of parameter solutions extracted from songbird neurons was done and is published in Nogaret et al. *Sci. Rep.* (2016): mean expectation value, variance and covariance.

The RVLM conductance model is a variant of the above models with same equations give or take one type of ion channel. It is an exemplar of the minimization problems common to all conductance models mentioned above. The particulars of any given model do not change the fact that measurement noise perturbs cost function minima (and therefore parameter estimates) in predictable ways. Note that the results of the first section derive from the property of the cost function itself and not of the choice of model.

We have now stated earlier successes of data assimilation in building quantitative models of biological neurons. We also explain the need to go back from biological data to model data to validate convergence. The following statement has been added at the beginning of the second paragraph of the introduction: “Here we address the problem of multi-valued solutions … or analog device equations.”

line 102: using a least-square error function has as big drawback that it is very sensitive to tiny shifts in timings. E.g. if an AP is shifted by a couple of ms, it's exact shape could still be the same, but the error could be huge. Could you discuss this in the paper, and how this could be solved.

REPLY 2: This is not a drawback, on the contrary. The least-square function assigns a large penalty to mismatches in the timing of action potentials between the model and the data. It therefore does objectively what it is supposed to do, which is to force the model to account for the precise timings of action potentials.

line 193: at the moment you're adding noise to the voltage. I'd also suggest adding noise to the current, which would reflect reality better and which could lead to some shift of e.g. the AP timings etc.

REPLY 3: The effect of adding noise to the injected current is completely negligible compared to adding noise to the membrane voltage (we have tried both). This is because the membrane capacitance integrates the current noise. Recall that the membrane voltage equation integrates the injected current through the first equation of the Hodgkin-Huxley model:

$$C\frac{dV}{dt} = \cdots I_{inj}$$

line 279: it's not very clear to me how this percentage was obtained. As I mentioned above, I also think these numbers are too precise and for a particular case, I'd rather see mean/std of these over a couple of trials, use cases.

REPLY 4: We have now clarified this point by rewriting: "Assimilations of the RVLM neuron model starting from 1000 random samples of parameters and state variables was found to converge to the optimum solution with a probability of 94.3% using noise regularization, and 67% without. In the other 5.7% and 33% of cases, convergence terminated at local minimum."

line 331: to say 'systematic' it has to be quantified better.

REPLY 5: Parameters taken at the local minimum nearest to the global minimum produce missing spikes in panels c) and d). The "systematic error" comes from the fact that the parameters of local minima situated further away from the global minimum will invariably yield worse predictions.
The key message here is that no *two distinct sets* of parameters can give the same perfect prediction. This result invalidates the claim made in the biological literature that a plurality of parameter sets can underpin a given time series recording of the neuron membrane voltage.

line 352: typo: missing function 'of' a biological

REPLY 6: corrected, thank you.

line 415: I think, based on the fact that this is very theoretical work, it is far too much a stretch to start draw conclusion from this about brain function.

REPLY 7: Of course, at face value, we agree with the above comment. However this image is helpful to soften the theoretical language of this paper which was mentioned by all 3 reviewers. This analogy to the brain now supported by an added reference helps make the dry theoretical conclusions more accessible to the audience in particular biologists.

line 515: about the adaptive time step approach in general. The NEURON simulator has an adaptive time step approach that does exactly this in a more advanced way (i.e. decrease the time step at times when more data points are necessary). It would make sense to reference and discuss that method.

REPLY 8: There are major differences in the mathematical formulation and purpose of the adaptive step size of forward integration algorithms (NEURON, 5th order Runge-Kutta, odeint() Python routine) and the adaptive mesh size of our variational method:

- In forward integration, the adaptive step size is needed to carefully navigate through the bifurcations of stiff nonlinear systems.
- The variational method we have developed is a *boundary value problem* with additional constraints that determine the continuity and smoothness of state variables. The purpose of the adaptive step size in this context is not to deal with system stiffness but to increase the length of the assimilation window and maximise the number of APs within it. This is driven by the need to increase the accuracy of parameters estimates at fulfil conditions of identifiability and observability.

To our knowledge, it is the first time an adaptive mesh size is implemented within the interior point method.

About the choice of parameters. You do tune a lot of parameters at the same time. Especially the kinetic parameters can in generally be obtained from separate experiments / literature. It might make sense to discuss if it's better to fit everything at the same time, or break things up in different components.

REPLY 9: Parameters as a whole are obtained as a solution of variational optimization. Unicity of the solution is warranted by Takens' embedding theorem. The correctness of estimated parameters is demonstrated in Table 2 and Fig.5. It is true that voltage clamp experiments may obtain some parameters, but most parameters are very difficult to access experimentally making parameter estimation methods central to the extraction of unobserved parameters. In addition, with voltage clamps, parameters need to be obtained from multiple cells and combined or averaged. Due to model nonlinearity the average or combination of parameters does not equal average cell behaviour.

Reply to Reviewer #2:

Overall this article would be a worthwhile addition to the field of neuroscience and touches upon the common problem of parameter estimation for nonlinear models from noisy data. The paper is split into clear distinct sections and the figures are quite useful to gain an intuitive feel for the methods used.

However, the authors should make their work slightly more accessible to a general audience and include more references to existing, different approaches to parameter estimation in the literature.

Furthermore, the scope and applicability of the methods should be more clearly outlined, such that other readers in neuroscience can readily see in what circumstances this research is relevant.

REPLY 10: Yes, we agree on this, and on the related point of Reviewer 1 (Reply #1). We ought to have better linked our choice of RVLM model to earlier successes in modelling biological neurons. We now re-establish this link in the second paragraph of the introduction: "Here we address the problem … analog device equations".

Sometimes the notation and the steps performed for each section are unclear. Nevertheless, these shortcomings should be relatively straightforward to address and does not require a radical overhaul of the paper.

In more detail:

Equation (1) – Are there any restrictions on the equations or the state variables for your approach? For example, can each state variable depend on all the other state variables, or are there restrictions on this?

REPLY 11: Any system of time dependent nonlinear differential equations may be assimilated using the interior point method. Our group and others have successfully assimilated actual neuron oscillations with both multi-ion channel Hodgkin Huxley-type models (Meliza et al, 2014; Nogaret et al, 2016; Vavoulis et al.,2012) and VLSI neuromorphic circuit equations to assimilate electrophysiological recordings of actual neurons (Abu-Hassan et al., 2019; Wang et al., 2017). This is now recalled in the introduction "… Hodgkin-Huxley type equations and analog device equations"

Our state variables represent distinct physical quantities hence are explicitly independent. Implicitly coupled state variables (e.g. two variables dependent on temperature) may also be assimilated but their implicit dependence adds one more constraint.

Line 49 – Presumably "pairs of parameter sets" is meant here rather than two parameters from a given set.

REPLY 12: correct, we have corrected this by writing “pairs of parameter sets”.

Line 103 – Make it clear that x(0) refers to an initial condition of the state variables for the model.

REPLY 13: we have added in the text that the “x(0) are the initial conditions of the state variable for the model”.

Lines 119-120 – The description of the experimental error should be more closely related to the biophysics of the problem. For neurons there is channel noise, thermal fluctuations, fluctuations in synaptic drive (if *in vivo*) along with measurement error coming from the equipment used to measure the neurons. Add some references to the different types of noise which are present in neurons and for electrophysiological measurement (e.g. Faisal, Selen, Wolpert, 2008: *Noise in the Nervous System*).

REPLY 14: We have related experimental error to the biophysics of the problem by adding “patch clamp noise, thermal fluctuations, stochastic processes associated with the opening and closing of ion channels, the binding of signalling molecules to receptors, and long term membrane potentiation”. The Faisal et al. reference is also included, thank you.

Line 121 – Presumably the additive noise at one time is independent from the noise at a different time. Briefly justify why this is a good assumption, as not all additive noise will necessarily have a white spectrum. Could your approach, in principle, accommodate temporally correlated noise?

REPLY 15: Yes, our approach can be extended relatively easily to account for temporally correlated noise and more generally temporally correlated measurements. This the least square cost function would be replaced with an objective function of the type:

$$\left(V_{exp} - V_{mod}\right) \boldsymbol{J}^{-1} \left(V_{exp} - V_{mod}\right)$$

where correlations between measurements are accounted by finite off-diagonal terms in the error conditioning covariance matrix $\boldsymbol{J}$.
Calculating such covariance matrices is very much the subject of ongoing research in meteorology – see for example Tabeart et al, The conditioning of least-squares problems in variational data assimilation, *Numerical Algebra with Applications* (2018) **25**, e2165.

Line 135 – Bold required for p_0^*.

REPLY 16: Done

Lines 147-148 – Add a further sentence for the general reader to intuitively explain the technical meaning of “sloppiness”, as I would not assume that the majority readers would recognise this.

REPLY 17: we have now added “The [curvature] term determines how tightly constrained a parameter estimate is, also labelled parameter ``sloppiness” by Gutenkunst et al.”

Lines 154-158, equation (6) – The noise entropy and this equation are not referred to afterwards in the paper, only two paragraphs before. Move this further up perhaps.

REPLY 18: the 3 short sections following equation 5 give comments on the 3 terms $H_{kk'}$, G_k and F. If one moves up the discussion of the F term (noise entropy), the discussion of the $H_{kk'}$ and the G_k terms would have to move down. This is a no win situation.

Line 175 – Why have you chosen a RVLM neuron model and not a different neuron model? As this is a test case for the methods you outline, explain what makes this model a good choice for this. This is touched upon in vague terms in lines 178-179 and later in the methods section, but you need to be more specific. The question that this explanation should answer to the reader is: what does your choice of model suggest about the applicability of your work to other neuronal models?

REPLY 19: the RVLM model is a variant of the conductance models of CA1, CA3 neurons, songbird neurons and respiratory neurons which we have built earlier (see REPLY #1) give or take one or two types of ion channels. The RVLM model is exemplar of the convergence issues raised by all conductance models when optimized with the interior point method. We could have chosen a conductance model with more ion channels which would have costed more computational time to navigate through more local minima without qualitatively changing the nature of the optimization problem.

Lines 184, 194 – The useful membrane voltage that you have generated is now referred to as V_mem rather than V_use. Make this consistent with previous notation or adequately explain why the notation has been changed.

REPLY 20: Well spotted, thank you! This is now corrected in the text.

Lines 188-190 – Will the code used to perform the parameter search algorithm for the RVLM model upon acceptance and publication of the paper? Also, it is worth outlining that your four examples seem broadly applicable to any parameter estimation method that uses the same cost function. If some aspects are specific to an interior-point solver approach, please briefly state that this is the case.

REPLY 21: The code used to perform the gradient descent is the open-source interior-point optimizer IPOPT (*https://github.com/coin-or/Ipopt*). In order to interface with IPOPT, it was necessary to customize a symbolic computation software to convert the model equations into the constraints of the optimization problem, that is obtain the Jacobian matrix from the linearized equations and generate the Hessian matrix of the cost function. This software was built in Python and can be found at (*https://pypi.org/project/pydsi*). The code, together with the code needed to forward-integrate model equations and code to perform statistical analysis of extracted parameters can of course be made available.

The interior point solver is well suited for optimizing time dependent systems of equations. Systems possessing both space and time dependence (e.g. the Navier-Stokes equations used in weather forescasting) are typically optimized with 4D-Var instead of IPOPT - see for example: *4D-Var* by Ross Bannister (www.met.rdg.ac.uk). The aspects we have discussed are valid for gradient descent methods in general and not just interior-point solvers.

Equation 9 – It is not explained before or immediately after what R is here (the number of realisations).

REPLY 22: "*R* is the number of noise realizations hence the statistical sample of parameter sets used to calculate the covariance matrix" has been added in the text.

Line 229 – Throughout this paper "magnitude", "noise intensity", "noise amplitude" and "noise level" are used seemingly interchangeably for sigma. Be consistent and choose one of these to refer to sigma throughout, if indeed sigma refers to the same quantity in each section.

REPLY 23: Good point. We have now replaced noise "magnitude" and noise "intensity" with noise "amplitude" in several places - highlighted in red.

Lines 234-235 – How can one know in general which local minimum is the closest to the global minimum when a multitude of local minima may exist? Is some sort of heuristic in the Euclidean parameter distance used for this?

REPLY 24: Yes – $||p - p^*||$ is the Euclidean norm as indicated in lines 334-335. We have further clarified this in the caption of Fig.5(a) by writing: "Value of the cost function at the site of local minima (purple/orange/blue dots) in the vicinity of the global minimum (red dot) plotted as a function of the distance to the global minimum defined by the Euclidean metric".

Line 241 - What is the meaning of a negative noise amplitude? Surely this cannot refer to the standard deviation of Gaussian-distributed noise, so what does sigma refer to in this case? Is it a constant scalar factor for the whole noise realisation?

REPLY 25: A negative σ here implies the same Gaussian noise probability distribution but the amplitude of the temporal realization of this noise has opposite sign. This is now clarified in line 241.

Lines 248-249 – Can one safely assume that p_\sigma\zeta^* always lies inside the basin of attraction of the noise-free optimal parameter set? Is this guaranteed by the jump in delta p and delta c?

REPLY 26: The local minimum $\mathbf{p}_0^l$ and global minimum $\mathbf{p}_0^*$ are surrounded by their own basins. These basins are well separated in parameter space otherwise the local minimum would not exist. Adding noise shifts the positions of these minima, $\mathbf{p}_{\sigma\zeta}^l$ and $\mathbf{p}_{\sigma\zeta}^*$, relative to one another. If $\mathbf{p}_0^l$ and $\mathbf{p}_0^*$ are *not too far from one another* initially, there is a strong probability (94%) that the jump in $\delta\mathbf{p}$ and δc will occur. This jump is therefore almost guaranteed to occur.

The probability of jump will of course be less certain for local minima further away from the global minimum. However recall that local minima further away from the global minimum are less problematic because they produce worse model predictions of the membrane voltage hence can more easily be pruned by the line parameter search filter within IPOPT. The problematic local minima are those in close vicinity to the global minimum which give almost perfect predictions. Line parameter filtering cannot easily distinguish these vicinal local minima from the global minimum – hence the importance of our noise regularization.

Line 257 – epsilon_z has not been introduced yet.

REPLY 27: ε_z is “a parameter of the HCN activation gate which gives the difference in recovery times between the half-open and fully open state of the gate” as now indicated in the text. This is the difference in recovery times in the ON and OFF states of the gate. This parameter is among those listed in Table 2.

Lines 278-279 – What does it mean for the method without regularization to converge 67% of the time?

REPLY 28: We have now clarified this by writing: “Assimilations of the RVLM neuron model starting from 150 initial samples of random parameters and state variables was found to converge to the optimum solution with a probability of 94.3\% using noise regularization, and 67\% without. In the other 5.7\% and 33\% of cases, convergence terminated at local minimum.”

Are there different initial conditions x(0) or initial parameter guesses?

REPLY 29: Both initial conditions x(0) and initial parameter guesses were random. The statistical sample was 1000. This is now stated in the manuscript as per the phrasing in the previous answer.

How many trials were performed for each case?

REPLY 30: 150.

Furthermore, why does the regularized method fail 6% of the time ?

REPLY 31: Each of the 150 assimilations were performed starting from the vicinal minimum using randomly initialised state variables. The 6% failure arise from initial conditions too far away from the solution.

Is there an obvious pattern to the failures?

REPLY 32: The likely reason is that assimilation starting from some initial conditions will terminate at local minima too far away from the global minimum to be effectively regularised. This is a difficult question in the general case however (see P vs NP complete problem).

Do you have a comparison of the average computation time for the method without regularization to the method with regularization? One may ask whether it is quicker just to run the simulation without additive noise multiple times in the hope that the optimal parameter set is reached at least once.

REPLY 33: Including noise regularization does not significantly increase the computation time.

Lines 319-320 – Presumably this is p_0^v for one particular noise realisation? Furthermore, p_0^v seems to represent the same concept as p_\sigma\zeta^* in the regularization section. The notation should be made consistent if this is indeed the case and the connection between these two sections in general should be made stronger.

REPLY 34: This is correct - we have renamed $\mathbf{p}_0^v$ as $\mathbf{p}_{\sigma\zeta}^*$ for clarity both in the text and Fig.5.

Line 339 – Minor point: Takens'

REPLY 35: Corrected

Lines 350-352 – This claim needs a reference.

REPLY 36: Further research into the claim of 35% to 55% observation of neuron loss in ageing show this originates from animal studies conducted in the 1950s which have since proven exaggerated as this did not consider the natural reduction in size of the brain. A more recent review estimates the loss of brain cell with age to a more conservative 2%-4%. This reference [*J. Chem. Neuroanatomy* 93, 2-15 (2018)] and the revised numbers are now quoted in the manuscript.

Line 380 – Elsewhere in the paper 94% probability was given for convergence.

REPLY 37: Corrected

Lines 380-382 – Do you have an intuitive idea on how small the experimental error would have to be in comparison to the additive noise used for regularization? It would be nice if this additive noise regularization could be applied to already noisy data. Briefly state why the task is "hopeless" when the errors are sufficiently large.
Sequential Monte Carlo (or particle filter) approaches have been used for parameter inference for nonlinear models from noisy neuron data (e.g. Huys and Paninski, 2009: *Smoothing of, and Parameter Estimation from, Noisy Biophysical Recordings* and Vavoulis, Straub, Aston and Feng, 2012: *A Self-Organizing State-Space-Model Approach for Parameter Estimation in Hodgkin-Huxley-Type Models of Single Neurons)*. How does your research relate to this?

REPLY 38: These are all good points. We have now described the limitation of our gradient descent method enhanced by noise regularization and compared it to particle filter approaches (and path integral approaches) in the section: "This method is well suited … whereas the present variational approach does not." Thanks for pointing to the above two papers which we have now included.
Our assimilation of early and noisiest songbird neuron data (Meliza et al., 2016) failed to converge when the noise level exceeded 3-4mV for windows with 50,000 to 100,000 data points. Membrane voltage time series had to be smoothed to arrive at sensible parameter estimates. Currently, state of the art patch clamp recordings now routinely achieve much lower noise levels < 0.5mV.
The cost function at the global minimum is several orders of magnitude below both its value at the local minima and the depth of local minima (see Fig.5a and Ye at al. PRE 2015). If 3-4mV is the experimental error that washes away the global minimum, we expect that a weaker noise level (e.g. 0-2mV, either experimental or additive) will facilitate convergence by weakening the local minima at the expense of the global minimum.

Line 381 – Typo: "their"

REPLY 39: Corrected.

Line 435 – Per area membrane capacitance Fig 3a - Is the panel drawn here merely illustrative of the method, or does the curve actual represent how the cost function varies according to a specific parameter from the set (if the latter, which parameter)?

REPLY 40: This is correct. Fig3a plots the profile of the cost function drawn along a straight line passing through the global minimum and a vicinal local minimum as is now clarified in the caption. This line is in general a linear combination of the parameters listed in Table 2. This is why the jumps in δc and $\delta \mathbf{p}$ (Fig.3b and 3c) are observed when any single parameter is varied.

Figure 3e – This panel is just two numbers, it seems a bit redundant.
Figure 5a – The blue dots are not explained or referenced in the text. Are there other noise realisations of p_0^v?

REPLY 41: the blue dots are now referenced in the caption of Fig.5a. These are the local minima in the cost function located further away from the global minimum as per our Euclidean metric. We have now added a note in the caption to clarify this.

Reply to Reviewer #3:
The paper Review Estimation of neuron parameters from imperfect observations discusses the model parameter estimation of single neurons based on observed membrane potential. The authors introduce key challenges, namely observability, identifiability, local minima and ill-definition. Further on, the authors discuss two sources of errors, experimental errors and model errors and how they influence the global and local minima of the cost function by second-order expansion. They discuss very detailed the effect of experimental error and model error on location and orientation of minima. As a solution, the authors suggest a new method by scaling the experimental noise, which moves and suppresses local minima, making it more likely to identify the optimal parameters of the model. They present numerical results based on RVLM neurons where they demonstrate the improvements in parameter estimation by their proposed new method.

As a second part, the authors discuss the requirement to drive the model by a various external current to cover the full range of dynamical behaviour of the model. The authors study the parameter estimation and their correlation by varying the number of data points. They suggest a method of non-uniform sampling the time series to optimize parameter estimation and de-correlation while not increasing the number of data points. Authors support this by numerical experiments.

In summary, the authors provide a very detailed and extensive discussion of the topic. The reach of the study is limited by the assumption that the model is known and only the model parameters p need to be estimated. In an experimental setting this would not be the case. It would help the understanding of the paper if the authors could mention this limitation.

REPLY 42: The relevance of the model and assimilation procedure to actual biological neurons is now discussed in the introduction as answered in REPLY #1. This was an omission on our part as we have published extensively on the construction of quantitative models of CA1, CA3, songbird and respiratory neurons. The motivation of this paper was to address the uniqueness of parameter estimates arising from the assimilation of actual neuron data.

It was not part of this study to optimise parameter estimation if the data generating model is not equal to the fitted model as it would be with experimental data. As far as I understand is the entire study based on the assumption that experimental noise is additive and uncorrelated. I believe the consequences of this assumption could be discussed in more detailed and maybe also the effect of different types of noise.

REPLY 43: We agree on both counts.
We did not perform a systematic numerical investigation of *model error* as Eqs 4 and 5 tell us that model error only gives a second order correction to the cost function. This is why we have focussed on the consequences of experimental error. The results of Eqs.4 and 5 is consistent with our experience of assimilating biological neuron data where HH-type conductance models are accurate enough to estimate threshold and kinetic parameters as evidenced by comparison with measured thresholds and recovery times.

The point of correlated noise vs uncorrelated noise has been raised by reviewer 2 above and answered in REPLY #15 above. Noise correlations would be accounted for by modifying the least square cost function with a conditioning covariance matrix accounting for correlations between temporal measurements in its non-diagonal terms.

To me, the section describing the noise regularization to improve convergence is a bit unclear: The text says a specific noise realization is selected and this this noise realization is scaled in amplitude. Does this not imply that the noise is already known. This section could be a little more detailed.

REPLY 44: The section on noise regularization shows that adding noise to experimental observations (possibly already incorporating experimental noise) is a regularization tool that can improve the convergence of the parameter search. We have clarified this further in the text. See also REPLY #38.

The manuscript is very clear and consistent when it comes to mathematical formulations. I believe the manuscript could benefit from an iteration of the text, especially for readers which are non-experts.
Overall, I think the paper covers an interesting topic in a very strict mathematical form. The manuscript could be improved by discussing the implications of the made assumptions as well as by improving the writing to make the text easier to understand and read for non-experts. Therefore, I recommend a minor revision.

I would like to mention some specific points which could be changed to improve the manuscript:

1. *Abstract*

The sentence "This regularization method increases the probability of finding the true parameter configuration from 67% to 94:3%." is unclear since it is not defined what the true parameters are. Later on it is mentioned that it is meant finding the global minimum, but this could be mentioned here as well to avoid confusion.

REPLY 45: We have now corrected this in the abstract by writing "the probability of finding the optimal set of parameters from 67% to 94.3%".

2. *Introduction*

Line 35: Would make it easier to understand if the L +K state would be explicitly defined as the parameter state

REPLY 46: *L*+*K* is the size of the problem within the interior point optimization method whereby *L* is number of state variables and *K* is the number parameters. Parameters are treated as state variables satisfying $\dot{\boldsymbol{p}} = 0$ as commonly done many optimization schemes (e.g. Vavoulis et al, 2012). We have made the appropriate rectifications (in red) in the text.

Line 71: Since the PDF is non Gaussian, it could be helpful to also use percentiles or something which could deal with heavy tailed or other distributions.

REPLY 47: The spectrum of central moments $\mu_n = \langle (p - \langle p \rangle)^n \rangle$ of the Gaussian distribution will vary from parameter to parameter and as a function of noise amplitude as shown in Fig.2. A theory for computing non Gaussian spectra is therefore needed to establish their general properties. In the absence of such theory, we would like to remain prudent about drawing general conclusions from the PDFs which we have simulated in a few cases.

Line 74: Define "surfaces of constant misfit"

REPLY 48: These are surfaces of constant “data misfit error” i.e. the ellipsoids defined as δc = constant (Eq.4). This has now been corrected in the text.

Line 81: Sentence hard to understand

REPLY 49: Sentence was rewritten as “For this we introduced an adaptive sampling rate which applied a smaller temporal step during action potentials than during intervals of sub-threshold oscillations”.

3 Results

Line 123: Should the model errors also depend on I(t)?

REPLY 50: Yes, one rigorously ought to write the model error as $\epsilon_{mod}(t, x(0), p) = V_{mod}(t, x(0), p, I_{inj}) - V_{use}(t, I_{inj})$ where both the model membrane voltage and the useful part of the experimental membrane voltage both depend on the *same current injection protocol* $I_{inj}(t)$. In aggregate ϵ_{mod} will remain largely independent of I_{inj} as any dependence will be cancelled by the difference $V_{mod} - V_{use}$.

Our argument hinges on the Taylor expansion of the data misfit error δc (Eq.4) with respect to parameters $\boldsymbol{p}$, and on the observation that V_{mod} depends on $\boldsymbol{p}$ whereas V_{use} does not. To avoid confusion we have deleted the reference to the dependence on **x**(0).

Line 139: The model has not been introduced, so this sentence is hard to understand. Also, what is exactly the sensitivity of the membrane voltage?

REPLY 51: We have replaced the previous phrasing with: “The dominant contribution to the $\partial V/\partial p_k$ term will come from the jumps in membrane voltage (-100mV <-> +45mV) that can be induced by minute changes in parameter values near action potentials.”

Line 159: What is principal analysis?

REPLY 52: We ought to have written “principal component analysis”. This is now corrected in the text.

Line 241: How is negative noise amplitude defined in context of normal distributions? Should σ not be larger than zero?

REPLY 53: A negative σ here implies the same Gaussian noise probability distribution but the amplitude of the temporal realization has opposite sign. This is now clarified in line 241 (see also REPLY 25).

Line 280: Why does a random input I not work?

REPLY 54: If the question is why does assimilation still fail to reach the global minimum 6% of the time under noise regularization, the answer is that parameter search may reach a local minimum too far away from the global minimum for the “jump” in δc and δp to occur when the noise amplitude is varied. Fig.5a (blue dots) shows the distribution of local minima. Also note the statistical sample of parameter sets used to obtain the 94% figure was 150 assimilation with random parameter and state variable initializations.

Line 250: The parameter search was initialized given the ground truth.
What happened if it was randomly initialized?

REPLY 55: the parameters are not initialized at the ground truth (global) minimum but at a local minimum. The state variables were initialized randomly. The 67% and 94% success rates were obtained from 150 random initial guesses of state variables. This is now clarified in the text.

4 Methods

Line 511: Units not consistent, if u is in volts, why is derivative of *u* unitless?

REPLY 56: The units of du/dt are: mV.ms^{-1} – this is now corrected in the text. Thank you for spotting this.
