# Supplementary material for: Estimation of neuron parameters from imperfect observations
Source: PLoS Comput Biol. 2020 Jul 16;16(7):e1008053. doi: 10.1371/journal.pcbi.1008053 (PMC7386621; doi:10.1371/journal.pcbi.1008053)
Supplement: Attachment — Submitted filename: reviewer2-second-review.pdf [file pcbi.1008053.s005.pdf]

Paper revision, second round

The authors have addressed the comments made well, and have answered the main scientific and methodological questions. My further comments therefore mainly address typographical and aesthetic changes.

Throughout the paper: Both the different error types and the recovery time difference parameters are denoted by subscripted \epsilon. In the figures, the parameters are denoted by \varepsilon. For clarity and consistency, it would be worth denoting the different error types by \epsilon and the recovery time difference parameters by \varepsilon (or vice versa).

Lines 134-135: I thank the reply (#15) to my question about temporally correlated noise in their approach. Could the authors explicitly (but briefly) state that the noise used is temporally uncorrelated, but add the explanation that the scheme can accommodate temporally correlated noise, as explained in Reply 15? This can be placed in the discussion if preferable. It would be an asset to the paper to inform the reader of the generality of this step.

Line 229: “noise amplitudes”

Line 236: “and hence”

Line 348: A remaining p_{\sigma\zeta}^v here to be converted to p_{\sigma\zeta}^*

Figs 1-5: Make all references to the various p in the caption bold as in the main text. Also apply to the figure if possible. Furthermore, make formatting of text in the figures consistent. p_0^l looks particularly odd here in comparison with the main text.

Fig 1: Parentheses missing in caption for \delta c = f(\sigma, \zeta).
